# Promoting Navigation Health Literacy at the Intersection of Schools and Communities. Development of the Game-Based Intervention Nebolus

**DOI:** 10.3389/fpubh.2021.752183

**Published:** 2021-11-17

**Authors:** Kevin Dadaczynski, Verena Krah, Demian Frank, Elisabeth Zügel-Hintz, Fabrice Pöhlmann

**Affiliations:** ^1^Department of Nursing and Health Science, Fulda University of Applied Sciences, Fulda, Germany; ^2^Centre for Applied Health Science, Leuphana University Lueneburg, Lueneburg, Germany; ^3^HelloDesign, Wiesbaden, Germany

**Keywords:** navigational health literacy, location-based games (LBGs), adolescents, schools, communities

## Abstract

Emerging empirical evidence indicates a limited health literacy for a substantial proportion of children and adolescents. Although it is generally agreed upon promoting health literacy as early as possible in the lifespan, there is a lack of interventions addressing children and adolescents and their primary living environments. This article describes the development of Nebolus, a game-based intervention aiming to promote navigation health literacy at the intersection of schools and communities. Its intervention foundation lies in a socio-ecological understanding of health as well as in the Entertainment Education approach. Following an extensive literature search on health-related location-based games, a co-creation process was initiated that involved adolescents, community stakeholders, and design/IT professionals in all phases of the intervention development. The final Nebolus intervention includes three core activities: (1) a Nebolus rally app for adolescents aged 12 to 16 years, (2) an online planning tool allowing local health service providers/professionals to set up own Nebolus rallies, and (3) accompanying teaching material on health literacy in the school setting to be used before and after the Nebolus rallies. This article provides an overview of the intervention layout and discusses strengths and challenges of its development and implementation.

## Introduction

According to Sørensen et al. (1) health literacy can be understood as a modern concept including the individual ability to find, understand, appraise and apply health information to restore, maintain or promote health in everyday life. International surveys conducted in recent years (2, 3) as well as the high amount of health information especially during the Corona pandemic (4, 5), highlight the increasing importance of health literacy for public health research, policy and practice. Although numerous empirical findings on health literacy for adulthood are now available, research in childhood and adolescence is still in its infancy. While several instruments have been developed in recent years to assess health literacy in younger age groups (6, 7), they have not yet been widely used. Recent findings from the Health Behavior in School-aged Children (HBSC) study revealed a medium or low health literacy for 80% of the respondents with the highest proportion found in Germany and Poland (both 87%) and lowest in Finland (62%) (8). In comparison, the proportion of adolescents with low health literacy in a cross-cultural comparative study varied by instruments between 23.7 and 45.5% (9). Furthermore, preliminary evidence on digital health literacy from Germany indicates that young people most often report difficulties in searching for and critically evaluating digital health information, at 42% each (10).

Health literacy has not only been linked with several proximal and distal health outcomes, but also with the use of healthcare services, the receipt of health screenings or adherence to non-medical and medical treatment (11–15). Given its high predictive power, it might be surprising that there is a lack of health literacy interventions for young people. In their review, Berkman et al. (16) examined the effects of health literacy interventions on health care service use and health outcomes. Most intervention studies included (*n* = 42) focused on adult patients with only four interventions also including patients younger than 18 years. Another recent review focused on health literacy interventions in European countries and was able to identify only one intervention targeting children between 8 and 12 years (17). In terms of non-clinical health literacy interventions for adolescents, we are aware of only a few school-based interventions. Mclucki et al. (18) report the effects of a Canadian high-school mental health literacy curriculum including six modules that are delivered by teachers in 10 to 12 h. Evaluation results revealed substantial improvements in mental health knowledge and attitudes. The German foundation “Gesundheitswissen” (19) developed another curriculum-based intervention called “Pausenlos Gesund” (engl: non-stop healthy). It aims at the promotion of general health literacy in secondary school children and contains seven overarching modules (e.g., Finding good information, How does our healthcare system work?), a knowledge-focused board game and an explain video. So far, no evaluation data on uptake by schools and effectiveness are available. The HealthLit4Kids program aims to improve the health literacy of the entire school community and includes four stages (needs assessment, discovery, action planning and evaluation) (20). These allow schools to develop a need-based action plan and to create and deliver classroom activities.

Against the background of the limited intervention basis, we developed a tailored-based universal health literacy intervention (called Nebolus) addressing adolescents at the intersection of schools and communities. The main intervention aim is to strengthen navigation health literacy of adolescents in their direct living environment (e.g., district, community). Specifically, this includes the ability to find, understand, assess and use information about and services provided by organizations or professionals in a person's vicinity. This article describes the intervention development including basic conceptual foundations and the methods used during the developmental process. Moreover, an overview of the intervention layout and the implementation strategy is given.

## Conceptual Foundations of the Intervention Nebolus

### Navigation Health Literacy

Problems in navigating through an increasingly complex health care system have been identified as a challenge for patient centered health literacy (21, 22). Based on a newly developed instrument [HL-NAV, (22)], most current results from a representative German population survey suggest that more than 80% have difficulties in navigating the health care information environment (23). Most navigation problems could be identified for understanding information about current health care reforms, in finding out what support options are available to help navigate the health care system or in finding information about the quality of health care providers and their services. Comparable to individual health literacy, navigation health literacy can also be understood as a relational concept with the health care environment and its structures contributing to individual capacities for orientation and navigation. Outside the health care system, navigation health literacy has not been sufficiently addressed so far. In universal prevention and health promotion, communities, districts and neighborhoods serve as the primary living environment for young people, with sport and youth clubs or counseling centers as prominent examples for non-clinical sub-settings (24). Findings on utilization of non-clinical health services among adolescents and their navigational barriers are scarce and have mostly focused on help-seeking in the area of mental health. Poor health literacy was identified as a major barrier to seek for professional support on mental health including lack of knowledge about help sources and the inability to recognize early signs of mental health problems (25–28). In their mixed-methods study, Wang et al. (29) identified knowledge barriers such as lack of knowledge about mental problems, support options and providers as disablers for school-based mental health help-seeking for Asian- and Latin-American adolescents. In turn, positive past-experiences with health services, the perception of supportive and understanding health service providers were found to be important in facilitating help-seeking (27, 30). Knowledge about the existence and availability of community health services, the ability to evaluate their quality and confidentiality, positive experiences with the providers, and the perception that these services promote or maintain one's own health can be seen as important determinants of utilization. Hence, based on the definition provided by Sørensen et al. (1), we understand navigation health literacy in the local context as the ability and motivation to find, understand, evaluate, and apply health-related information and services provided in or by organizations or professionals in a person's vicinity (e.g., city, district, neighborhood).

### Health Literacy as Part of Comprehensive School Health Promotion

**Figure 1:**
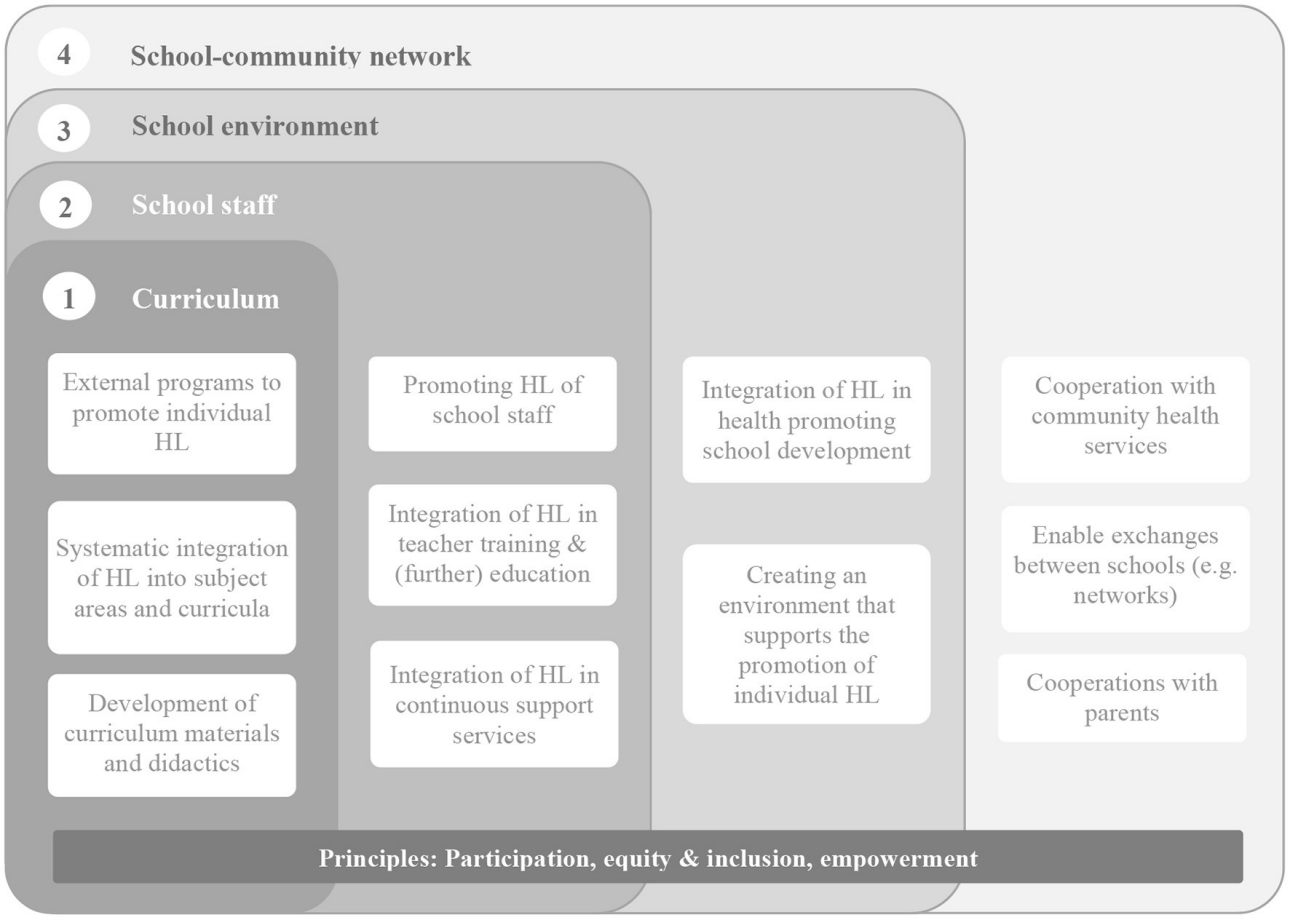
Health literacy as part of a holistic concept of Health Promoting Schools (31, 35).

Schools as community embedded systems have long been identified as an important avenue for health promotion and nowadays also for health literacy. First, as health knowledge and behavior are already established in the early phases of the life course, activities on health promotion and health literacy should start as early as possible focusing on primary living environments such as schools. Second, health literacy is compatible to the education core mission of schools and share many commonalities with existing curricular requirement and programs (31, 32). Third, schools can also have an influence on health and health behavior through their structures, conditions and processes (33). Fourth, schools provide young people an inclusive and equitable access to education as a key determinant of health regardless of their socioeconomic, cultural or political background. This is especially important as empirical findings suggest, that health literacy follows a social gradient with higher frequencies for sufficient health literacy found for young people with higher family affluence (8). Compared to singular often pre-packed interventions that focus on individual behavior alone, holistic interventions that also address the physical and social environment and consider all members of the school and the wider school community are thought to have a higher potential for impact. In order to avoid competition and conceptual confusion for schools it has been suggested that novel approaches such as health literacy should be integrated in the holistic framework of the Health Promoting School (HPS) approach (34). Figure 1 offers such an integrated perspective that is highly compatible with the HPS approach and its underlying values (e.g., participation, equity, inclusion). On a school class level, health literacy should be adressed by subject-focused and cross-curricular programs and interventions [e.g., (18, 19)]. In light of the growing body of research on health literacy among teachers (36) and school administrators (37), the promotion of health literacy should also focus on school staff. This is important not least because educators have a critical role in teaching health literacy to pupils or act as important agents of organizational change. Following the concept of health literate organizations (38), a health literacy friendly school environment should be created (e.g., as part of the ongoing school development processes). Finally, as schools are first and foremost educational organizations, strong intersectoral collaborations with community-based health professionals and their services are needed to promote health literacy. This also includes cooperation with parents and exchange of knowledge and experiences between schools.

### Entertainment Education and Gamification as Innovative Intervention Approaches on Health Literacy

In light of study findings indicating a higher frequency of limited health literacy for those of lower socioeconomic status, the intervention focus should be on those vulnerable groups from which we know that are hard to reach. In particular, traditional forms of information provision and communication, which are cognitive and rational, reach their limits when it comes to targeting groups with the greatest need for prevention (39). In the German discussion, the term “prevention dilemma” has been coined to emphasize the disproportionately high level of participation in traditional, often behavioral, prevention and health promotion interventions among low-risk target groups which increases the probability to widening the health inequality gap (40). From communication research the elaboration-likelihood model (ELM) can be used to explain differences in information processing (41, 42). When individuals show high motivation and (cognitive) ability to process information that are perceived as important, they will carefully examine the information and arguments which will result in more stable (health behavior) changes. In turn, individuals with low motivation and (cognitive) abilities are more likely to process information in a less effortful way by assessing simple social cues or heuristics (e.g., credibility of the statement, attractiveness of the communicator, length of a message). Compared to the “central route” health persuasive effects resulting from the “peripheral route” will be less likely to be stable. Chiang and Jackson (43) argue that individuals with high levels of health literacy are more likely to process (health) information in a cognitive careful way (central route), while those with limit health literacy tend to examine (health) information more often by peripheral cues and heuristics. The development of public health interventions should take into account the relationship between health literacy and information processing, i.e., there is a great need for interventions that go beyond the provision of information by also focusing on appeal and emotionality, both in terms of content and formal design. Entertainment Education (EE) is such a fruitful approach which can be defined as a communication strategy that uses popular media such as film, music or other new media to distribute prosocial (e.g., health-related) messages (44). Entertainment Education is usually characterized by an engaging story, that allows the audience to be absorbed into new worlds and appealing characters. Empirical evidence suggests small but significant effects of EE on health knowledge, attitudes, intentions, and behaviors (45). Compared to TV or radio serials, video or online games are a relatively new way to communicate health information and messages. While the term gamification refers to “use of game design elements in non-game contexts” [(46), p. 10] serious games are characterized to be intertwined with an educational approach by imparting knowledge or skills (47). A recent state-of-the-art review examined 1,743 health games released between 1983 and 2016 in 23 countries (48). Most frequently used game types were puzzle games, casual games or simulations and most games could be completed within 60 min. Findings regarding effectiveness were mixed with most promising results found for physical activity (especially through exergames), for dealing with chronic diseases (49, 50) or for psychotherapy (51).

## Methods

Based on the background and theoretical foundation presented, the development of the Nebolus intervention was carried out in two consecutive steps: (1) literature search on health-related location-based games, (2) participatory-based development of the intervention Nebolus.

### Literature Review on Location-Based Games for Health

As emphasized before, video or online games can be regarded as a promising intervention approach that delivers health-related messages in a low-threshold way by also focusing on peripheral cues and heuristics such as an immersive story, appealing characters and game mechanics. However, the evidence is heterogeneous, with some studies showing only small to zero effects of health related videogames (52). In addition to the relatively short duration of many game interventions, it might be problematic to assume that skills acquired in the virtual world can be easily transferred into real world action (39). Therefore, games that work at the interface between digital and analog worlds and enable real-world experiences through digital media could reduce this gap. Location-based games (LbG) are a relatively new game genre that became extremely prominent with the release of Pokémon Go in 2016. Most importantly, compared to classic videogames, location-based games operate in a physical environment such as public spaces (parks, neighborhoods). In a more general approach, Leorke [(53), p. 38] defines LbG as any game that “[…] incorporates the player's physical location and/or actions in an outdoor or public space into the game via a networked interface.” Network interfaces refer to a digital device (e.g., smartphone) that allows to track the movement of the player in real-time using a Global Positioning System (GPS).

Against the background of the digital progress and the availability of location-based services (e.g., Google Maps, Mapbox), LbG's are becoming increasingly interesting for public health. Therefore, the goal of the literature review was to provide an initial overview of the thematic issues, potential effects, and implementation experiences of health-related LbG's among young people. To gain insight into the existing field of research an extensive literature search was conducted using Cochrane Library, EBSCOHost, EMBASE, ERIC, Medline PubMed und Web of Science Core Collection. In addition, a hand search of relevant journals was performed (e.g., Games for Health Journal, JMIR Serious Games, JMIR mHealth and uHealth). Eligibility criteria and search terms were defined using the PICO scheme (54): (1) Population: adolescents and young adults aged 13 to 29 years, (2) Intervention: all interventions using location-based games that addressed any determinant of health according to the socio-ecological model of health (55), (3) Comparators: in order to include a wide range of studies, no specific comparators were defined, (4) Outcome: next to proximal outcomes such as knowledge and attitudes, intermediate and distal outcomes (e.g., behavior, prevalence, morbidity) with reference to any determinant of health according to the social-ecological model of health (55) were considered. In addition, publication year (01/2010 to 09/2019), language (English or German), and study type (intervention studies, observational studies) were used as inclusion criteria. Following a stepwise selection process (title, abstract, full test) a total of 33 publications were included in the analysis. More than half (*n* = 18) were published in 2016 and 2017 with most coming from the U.S. (*n* = 14). In terms of the topics addressed, LbG's with focus on health behavior such as physical activity predominates. Twenty-six of 33 publications included examined aspects of Pokémon Go. In their review and meta-analysis, Khamzina et al. (56) summarize the findings from 47 studies including more than 33,000 participants. Results indicate that Pokémon Go players engaged in less sedentary behavior and increased their daily physical activity by 1,446 steps on average. By contrast, aspects of mental health were examined much less frequently. Ronen et al. (57) report the results of a treasure hunt LbG played by first year university students during the orientation week in groups. Compared to a group of non-players, a higher psychological well-being (better sense of belonging, orientation, higher level of peer relations) was found for students who participated in the LbG. In addition to topics with a direct link to health, indirect links to or determinants of health were also examined by some studies (e.g., nature experience, connectedness with nature, perception of urban spaces). Overall, however, no explicit relation to health literacy and its subdimensions could be found in any of the included studies.

### Co-creation Using the Living Lab Approach

To allow that health literacy interventions are tailored to the needs of the target group, a co-creation design method was used. Despite different terms and understandings, co-creation refers to a process that systematically involves those for whom a health intervention is to be developed (58). Co-creation has its roots in participatory intervention design, that goes beyond lower levels of participation (often called as tokenism) such as information provision or singular consultation (59). We used the so-called Living Lab (LL) approach as one method of co-creation. According to the European Network of Living Labs, LL can be defined as a user-centered, open innovation ecosystem-based co-creation approach, aiming to integrate research and innovation processes in real life settings (60). The core elements of the LL include:

- User engagement, i.e., active participation of potential users at all phases of the process
- Multi-stakeholder participation, i.e., involvement of representatives of the public and private sector who are relevant for the innovation or product to be developed
- Co-creation, i.e., a process that substantially alters the role of users and stakeholders from subjects of research to equally contributors of the innovation
- Real-life setting, i.e., all co-creation activities take place in real-life environments (e.g., schools, communities) to better illuminate the context for which the innovation is being developed

Living Labs can be organized in three phases of innovation development: (a) understanding the current state and identifying needs of potential users (Exploitation), (b) developing a prototype (e.g., a minimal viable product) including feedback loops (Experimentation), and (c) evaluating the potential impact and added-value of the innovation (Evaluation). Living Labs have been established in different fields of public health such as alcohol prevention (58) or primary health care (60, 61). In their recent integrative review, Kim et al. (62) could identify 15 studies reporting their LL experiences. The majority (*n* = 14) were conducted in Europe with older adults as the main target group. While the topics addressed ranged widely (e.g., monitoring daily life, fall prevention), all LL applied a multi-method approach (e.g., by including quantitative and qualitative methods of data collection) and were embedded in a real-life setting.

The main users of the Nebolus intervention are adolescents aged 12 to 16 years, which were actively involved from the very beginning by the establishment of a youth council. Activities included several interactive workshops in which local providers of health services, barriers of utilization and potential strategies how to overcome these were discussed and a game story and potential types of game characters were developed (Table 1). Later stages of participation included various user-testing scenarios and feedback-loops. On the level of stakeholders, we established an advisory board consisting of local health promotion and prevention providers and professionals. Activities included regular meetings where key features and implementation strategies were discussed and developed. Next to public health experts, we worked very closely with IT professionals on the design as well as the technical development and implementation (including UX designer, web developer).

**Table 1:** Co-creation of Nebolus using the living lab approach.

**Phase**	**Group involved**	**Activity**
Exploitation	Adolescents	•Creation of mental maps with local health providers and their services •Discussion of help seeking/health service usage and their barriers •Discussion of strategies how to overcome these barriers
Experimentation	Adolescents	•Development of a game story and types of game characters •First look and feel of the Nebolus app click prototype incl. feedback discussions •First MVP testing of the Nebolus app incl. feedback discussions
	Health experts/stakeholders	•Regular discussions of key features for planning a LbG and its local implementation •First look and feel of the Nebolus planning tool click prototype incl. feedback discussions •Development of a first case scenario for the implementation of Nebolus in various communities
	UX Designer	•Iterative development of mockups •Iterative development of design assets and a click prototypes for the Nebolus app and the Nebolus planning tool
	IT Developer	•Iterative development of the Nebolus app •Iterative development of the Nebolus planning tool
Evaluation*	Adolescents	•Experiences of using the Nebolus App •Effects of the Nebolus app on navigational HL, help seeking attitudes and the intention to use local health services
	Health experts/stakeholders	•Experiences of using the Nebolus planning tool •Experience of working with other local stakeholders to develop/implement a local LbG

IT, information technology; MVP, minimal viable product; UX, user experience. *In planning.

## Results

### Objectives and Intended Outcomes of Nebolus

As described above, the Nebolus intervention aims to strengthen the navigation health literacy of adolescents aged 12 to 16 years in their direct living environment (community, district, neighborhood). This includes promoting the ability to (1) find information about local health service providers/professionals and their activities, (2) understand the services and activities provided by local health stakeholders and professionals, (3) evaluate the quality of local health service providers/ professionals and their offerings, and (4) apply health information obtained through local health service providers and professionals. The secondary objective of the Nebolus intervention is to promote intersectoral collaboration of health service providers/professionals and to stimulate the development of a coordinated prevention strategy at the community level.

To achieve these objectives, the intervention Nebolus pursues three core activities: (1) Implementation of Nebolus rallies in the local community for adolescents, (2) Tailored-based development of Nebolus rallies by local health service providers/professionals and (3) Implementation of accompanying teaching material on health literacy in the school setting to be used before and after the Nebolus rallies (Table 2, intervention actions). According to outcome models, different types of proximal and distal outcomes can be distinguished, which build on each other and unfold gradually. When designing the intervention, we used the outcome model of health promotion (63) to derive proximal and distal outcome assumptions based on the available evidence. As depicted in Table 2, we assume that Nebolus rallies and the accompanying teaching material lead to direct health promotion outcomes, which are improvements in (navigation) health literacy and positive attitudes toward local health service providers/professionals and their offerings. With respect to the subdimensions of navigation health literacy, we expect Nebolus to strengthen, in particular, the ability to find and understand information about local health service providers/professionals and their offerings which can be seen as a prerequisite to make use of them when needed. Moreover, we expect improvements in attitudes toward help seeking and health service utilization. These effects are expected to favor intermediate outcomes such as utilization of local health promotion/prevention services. With increasing utilization, a decrease in the prevalence of unhealthy behaviors and health problems of adolescents is expected at the distal outcome level. As shown in the right column of Table 2, it is intended that Nebolus LbG rallies are developed by local health service providers/professionals based on their public health needs and community infrastructure. This should result in strengthening existing or establishing new local networks (direct health promotion outcomes) and the development of a coordinated local health promotion/prevention strategy (intermediate health outcomes).

**Table 2:** Outcome model of the Nebolus intervention.

** 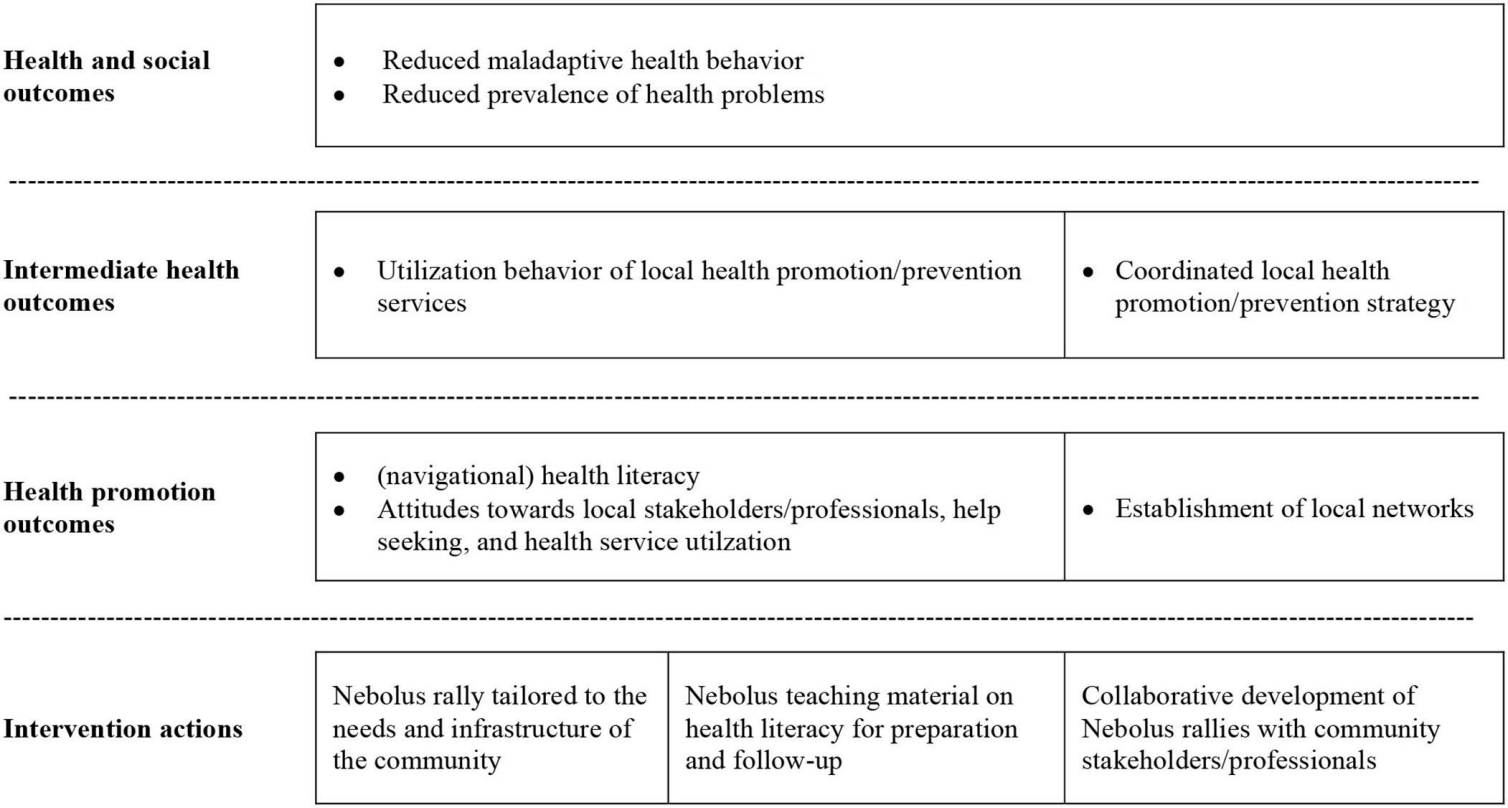 **

### Nebolus Rallies for Adolescents

**Figure 2:**
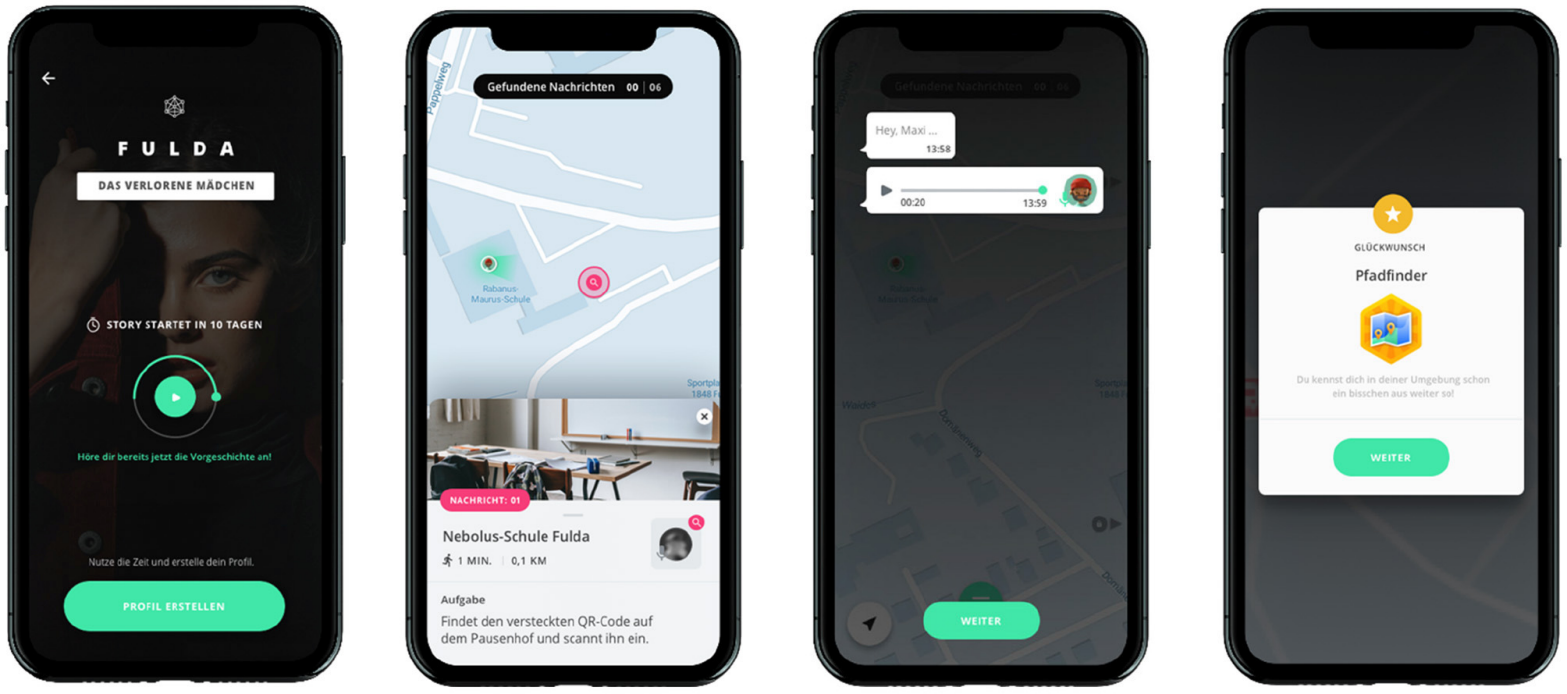
Impressions of the Nebolus LbG app.

The core of the Nebolus intervention is an app-based rally that guides young people to various real-world locations (stations or levels) in their respective community (Figure 2). At each station, users meet local health stakeholders/professionals and get in contact with the staff and their health-related services in a low-threshold way. Following the Education Entertainment (EE) approach, each Nebolus rally is based on an engaging and immersive story that is tailored to the local needs and interests. Stories are fictional and can serve different genres (e.g., crime and mystery, fantasy). Each story is presented via voice messages from the perspective of a main character and a close friend or relative. Voice messages appear before each station (with tips on how to find the station) and after completing the station (relevance of the station from the main character's perspective). At each station, health service providers and professionals pick up the thread of the story and integrate into it their organization and health-related services. Once they have interacted with the local stakeholder/professionals, users receive a QR code which unlocks further stations. To increase motivation, there are also so-called hidden places that only become visible on the map when the users are within a defined radius of this station. Moreover, various gamification mechanics are used. In addition to a sequence of levels (each station represents one level), a gender or cultural sensitive avatar representing the user in the game can be chosen and different badges can be earned depending on the progress (e.g., number of stations found, number of voice messages listened to). A progress bar graphically visualizes the progress of the rally (ratio of completed stations to the total number of stations).

Nebolus rallies are organized in groups of up to five users. This aims to reduce barriers to interaction with local health service providers and professionals as well as reduce the risk of exclusion of adolescents without access to digital media (smartphone).

### Tailored-Based Development of Nebolus Rallies by Local Stakeholders/Professionals

**Figure 3:**
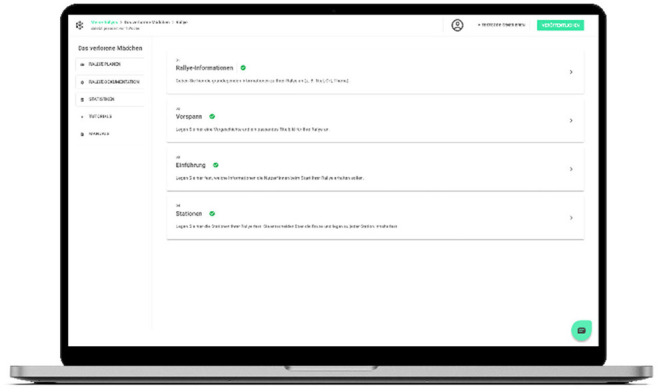
Impressions of the Nebolus planning tool.

Nebolus is a universal health literacy intervention that can be tailored to any specific health topic and the local context of the community. To enable tailoring, a browser-based Nebolus planning tool has been developed that allows each local stakeholder/professional to develop an own Nebolus rally. It is intended that a local stakeholder (e.g., local health authority, sport and youth club or a school) will take responsibility and coordinate all activities to develop and implement the local Nebolus rally. This includes the recruitment of local health service providers/professionals, the establishment of a local working group with all participating stakeholders, the development of a fictional story and to set up the rally using the online planning tool. To support communities to implement Nebolus on a local level a number of accompanying materials have been developed including checklists, a manual that helps to develop an own fictional story and characters, and a guide for local stakeholders to develop ideas for interaction with adolescents along the different dimensions of navigation health literacy. In addition, a YouTube channel was created, on which tutorials and explain videos will be made available successively. The Nebolus planning tool allows to set up a rally in four steps and does not require any specific IT related skills (Figure 3):

- Definition of basic properties for each rally, e.g., title of the rally, location, start and end date, main and secondary character
- Introduction into the rally that can be seen by the users before the start of the rally including e.g., a short description, a voice message presenting a background story
- Onboarding setup that can be seen by the users with the start of the rally (i.e., definition of several screens presenting the Nebolus rally and the tasks to be performed by the users)
- Creation of main and hidden stations including station name and description, address, text instructions for the users, and voice messages that appear before and after the station

For each Nebolus rally a unique rally-code will be created that needs to be entered in the Nebolus app by the users. This is to ensure that each rally is perceived as a unique event, without rallies from other communities being visible. However, each local rally will be documented as a case study on the Nebolus website (www.nebolus.net) and will serve as inspiration for other communities to develop their own rallies. Each case study will include information about the health topic addressed and the target audience, the story-framework and characters developed and used, the number of stations and local health stakeholder/professional, and information on how the rally has been implemented.

### Nebolus Teaching Units

Cross-curricular teaching material can be used by teachers to introduce health literacy before and to reflect on experiences and learning outcomes after the Nebolus rallies. Two preparatory lessons and two follow-up lessons are currently being developed, each with a duration of 45 min (i.e., 180 min in total). Each lesson includes background information for teachers, concrete learning objectives, a timetable, didactic instructions and accompanying material (e.g., worksheets).

The aim of the preparatory lessons is to introduce the concept of health literacy and to strengthen individual skills in the HL subdomains. The first lesson addresses the ability to search and find health information and includes group discussions about different information sources and their use as well as worksheets and group exercises about forms of information acquisition. The second lesson deals with the ability to critically reflect on and evaluate health information obtained through various sources. Specific focus will be given on digital health information and particularly on how to deal with information retrieved from social media.

In follow-up lessons, pupils are encouraged to develop a mental map of their local community that includes all local health stakeholders/professionals that were visited as part of the Nebolus LbG rally. This forms the basis for discussions within small groups and the class as a whole, for example, about the services offered by local health stakeholders/professionals and the experiences made with these by the fictional character of the Nebolus rally. In addition, barriers to utilization of these local health services (including the information that is provided by local stakeholders) and ways of overcoming them will be discussed.

## Discussion and Conclusion

As a result of increasing evidence, a German national action plan for health literacy was adopted under the auspices of the Minister of Health in 2018. It comprises a total of 15 recommendations across four suggested areas of action (64). Particular importance is attached to the education system, which, according to the recommendations, should be enabled to promote health literacy early in life. In addition, the action plan calls for community actions that provide residents with easy access to health information and strengthen their health literacy in collaboration with community stakeholders.

Due to a lack of interventions in childhood and adolescence, Nebolus aims to promote health literacy of adolescents aged 12 to 16 years at the intersection of schools and communities. It addresses two major recommendations of the German national health action plan and–through its focus on navigation health literacy–also provides references to two additional recommendations (#7: Facilitate navigation of the healthcare system, #8: Promote communication between health professionals and users). In addition, the Nebolus intervention allows for an explicit link to the Health Promoting School approach as it addresses the curriculum level and the school-community network. Through its focus on strengthening cooperation with community health services, Nebolus also contributes to intersectoral collaboration in school health promotion (65, 66).

Nebolus is characterized by several strengths: First, compared to traditional pre-packaged interventions, Nebolus provides an open intervention framework that can be adapted to the specific thematic and local needs. It therefore contributes to a shift from “one-size fits all” measures to targeting and tailoring health promotion and prevention (67, 68). Second, the development of the intervention is rooted in a co-creation process that involved youth, community stakeholders, and design/IT professionals from the very beginning. The iterative approach was intended to ensure that the needs of the youth and feasibilities of the local stakeholders were taken into account. Third, Nebolus explicitly addresses the relationship between health literacy and information processing. Based on the Entertainment Education approach, a low-threshold communication strategy including various gamification elements (e.g., story, avatars, level, badges) is applied. In contrast to informative/educative measures, health is addressed in a casual manner, which should lead to a higher motivation to participate, especially among those adolescents with limited health literacy.

Next to these strengths, several challenges need to be mentioned with regard to the intervention development and implementation: First, not all dimensions of the HPS framework (as depicted in Figure 1) are addressed by the Nebolus intervention. Given empirical findings indicating a low health literacy of educators and its association with mental health (36, 37), there is a need for promoting health literacy among school staff. Those activities should also focus on attitudes and teaching abilities as evidence from Taiwan could show that teacher's health literacy teaching beliefs, their attitudes toward health literacy instruction, and their level of confidence in their ability to teach health served as predictors for health literacy teaching intentions (69). As Nebolus is an intervention with a comparatively short duration, it can serve as an entry point to other long-term activities. This requires linkage with existing interventions, that focus on health-literate school and/or community development (20, 70, 71). Second, the open character of Nebolus requires action to be taken at the community level to adapt the intervention to the local needs and structure. Although a number of supporting materials are provided for this adaptation process, it can be assumed that communities are at different stages of their health literacy development. Therefore, using proven approaches such as the Community Readiness Model, specific forms of support tailored to the stage of development are needed (35). To make it easier for community stakeholders to start using Nebolus, story-frameworks on various health topics including the characters are currently being developed. These can be used by the community stakeholders and adapted to their own local needs. Finally, the current Corona pandemic poses a significant challenge to the implementation of Nebolus. In addition to the difficulty of adhering to infection control rules (e.g., sufficient distance when visiting local health services), school participation in Nebolus may currently be lower. Because of the learning gap, schools may tend to invest their time primarily in teaching core subjects, while health literacy receives little or no attention. Here it is important to emphasize that health literacy shares many communalities with existing curricular requirement (e.g., media literacy) and is not just an outcome but can also serve as a predictor for school achievement and school quality (32, 72).

In summary, Nebolus offers an innovative generic intervention framework that has the potential to strengthen (navigation) health literacy in adolescence. With the launch of the intervention, a number of studies are planned to evaluate the impact and the implementation process of Nebolus. The quantitative arm of the evaluation is planned as a cluster randomized trial, with schools within a given community serving as unit of randomization. Moreover, a qualitative evaluation arm includes interviews and focus groups with adolescents and local stakeholders/professionals about the experiences and facilitators and barriers during the implementation. In compliance with the Living Lab approach, active participation of all groups involved is planned during the evaluation.

## Data Availability Statement

The original contributions presented in the study are included in the article/supplementary material, further inquiries can be directed to the corresponding author/s.

## Author Contributions

VK and EZ-H conducted the scoping review. KD wrote the first version of the manuscript. All authors contributed substantially to the intervention development and contributed to manuscript revision, read, and approved the submitted version.

## Funding

This research was carried out within the eHLastic project (e-based health literacy at the intersection of schools and communities), funded by the German Federal Centre for Health Education on behalf of the Federal Ministry of Health.

## Conflict of Interest

The authors declare that the research was conducted in the absence of any commercial or financial relationships that could be construed as a potential conflict of interest.

## Publisher's Note

All claims expressed in this article are solely those of the authors and do not necessarily represent those of their affiliated organizations, or those of the publisher, the editors and the reviewers. Any product that may be evaluated in this article, or claim that may be made by its manufacturer, is not guaranteed or endorsed by the publisher.
